# Feasibility, acceptability and initial outcome of implementing community scorecard to monitor community level public health facilities: experience from rural Bangladesh

**DOI:** 10.1186/s12939-020-01265-6

**Published:** 2020-11-02

**Authors:** Shehrin Shaila Mahmood, Sabrina Rasheed, Asiful Haidar Chowdhury, Aazia Hossain, Mohammad Abdus Selim, Shahidul Hoque, Abbas Bhuiya

**Affiliations:** 1 Health Systems and Population Studies Division, icddr,b, 68 Shaheed Tajuddin Ahmed Sarani, Mohakhali, Dhaka, 1212 Bangladesh; 2 IMPACT study, ARK Foundation Gulshan, Dhaka, 1212 Bangladesh; 3 School of Health Sciences and Social Work, University of Portsmouth, Portsmouth, UK

**Keywords:** Community scorecard, Feasibility, Acceptability, Community engagement, Community clinic, Accountability

## Abstract

**Background:**

Engaging communities in health facility management and monitoring is an effective strategy to increase health system responsiveness. Many developing countries have used community scorecard (CSC) to encourage community participation in health. However, the use of CSC in health in Bangladesh has been limited. In 2017, icddr,b initiated a CSC process to improve health service delivery at the community clinics (CC) providing primary healthcare in rural Bangladesh. The current study presents learnings around feasibility, acceptability, initial outcome and challenges of implementing CSC at community clinics.

**Methods:**

A pilot study conducted between January’2018-December’2018 explored feasibility and acceptability of CSC using a thematic framework. The tool was implemented in purposively selected three CCs in Chakaria and one CC in Teknaf sub-district of Bangladesh. Qualitative data from 20 Key-Informant Interviews and four Focus Group Discussions with service users, healthcare providers, and government personnel, document reviews and meeting observations were used in analysis.

**Results:**

The study showed that participants were enthusiastic and willing to take part in the CSC intervention. They perceived CSC to be useful in raising awareness about health in the community and facilitating structured monitoring of CC services. The process facilitated building stronger community ownership, enhancing accountability and stakeholder engagement. The participants identified issues around service provision, set SMART (specific, measurable, attainable, relevant and time-bound) targets and indicators on supplies, operations, logistics, environment, and patient satisfaction through CSC. However, some systematic and operational challenges of implementation were identified including time and resource constraint, understanding and facilitation of CSC, provider-user conflict, political influence, and lack of central level monitoring.

**Conclusion:**

The findings suggest that CSC is a feasible and acceptable tool to engage community and healthcare providers in monitoring and managing health facilities. For countries with health systems faced with challenges around accountability, quality and coverage, CSC has the potential to improve community level health-service delivery. The findings are intended to inform program implementers, donors and other stakeholders about context, mechanisms, outcomes and challenges of CSC implementation in Bangladesh and other developing countries. However, proper contextualization, institutional capacity building and policy integration will be critical in establishing effectiveness of CSC at scale.

## Background

Engaging communities in local-level planning and management of healthcare delivery has emerged as a strategy to increase health systems responsiveness to the needs of its users. Community-based monitoring supported by social accountability principles has been used for improving quality of services and increasing the uptake of services in some countries [1, 2]. Consumer charters, village health committees, citizen report cards, community scorecards (CSC) etc. are a few notable approaches of instilling social accountability into monitoring mechanism of health facilities [3]. These social accountability approaches have been found to induce intermediate changes like improved transparency, more efficient use of resources, empowered citizens, improved perceptions of health services, which result in better health outcomes [4, 5]. Particularly the community scorecard has been tested in regions where weak management and governance, lack of accountability, absent or weak social contract between state and its citizens, and conflicts have plagued public services. However, to our knowledge the understanding around community scorecard implementation is still limited in developing countries. Moreover, for Bangladesh the use of community scorecards in health is rare.

In 2017, icddr,b aimed to develop and contextualize a community scorecard to monitor performance of community clinics (CC) which provide primary healthcare at community level in rural Bangladesh and maintain referral linkage with higher level facilities. Community clinic is a flagship programme of the current government of Bangladesh and in a country with over 100 million rural populations each community clinic is designed to cover 6000 population. The CCs are built on land donated by the community and are designed to deliver services in partnership with the community. The government provides the infrastructure, healthcare providers, medicines and other supplies and the community is responsible for maintenance, security and other costs. Till date, 13,500 CCs have been established in the country [6, 7]. Despite the widespread establishment of these clinics, the goal of ensuring quality, equity and accountability in healthcare service at the local level is limited by lack of supply and logistics, proper monitoring, and overall lack of voice and accountability [8]. At the same time, staff absenteeism and inefficient use of the supplied medicine by the CC staff add to these challenges. Given the potential of community scorecards in resolving these issues in other similar context, the tool was implemented at CCs.

With the growing global interest around potential of social accountability tools in accelerating progress in health it is essential that we document and learn from implementation experience of such tools, particularly in health systems faced with multifaceted challenges of accountability, governance, inefficiency in resource use and lower social involvement in health. The current study, therefore, presents learnings from implementation of community scorecards in rural Bangladesh in terms of its feasibility and acceptability. The paper also shares outcome of the scorecard and some potential challenges in implementation at selected CCs in rural Bangladesh.

## Methods and materials

### Management of community clinics

Each CC is managed by one 13–17 member Community Group (CG) and three Community Support Groups (CSG), each with 13–17 members (one-third female members). The CG is responsible for monitoring of health service provision at community clinics and for fund generation at community level. The CSGs supplement the work of CGs in managing community clinics and raising community awareness [7]. For the purpose of the current study, CG was taken to represent the provider group and will hereafter be referred as the “provider group” and the CSG was taken to represent the community and from hereafter will be referred as the “community representative group”.

### The community scorecard and its implementation process

Community scorecard at community clinics was implemented through the community and provider representatives from the clinic management groups (i.e. the CGs and the CSGs), with technical support from icddr,b project personnel. Three project personnel facilitated the whole process at the intervention clinics.

**Fig. 1:**
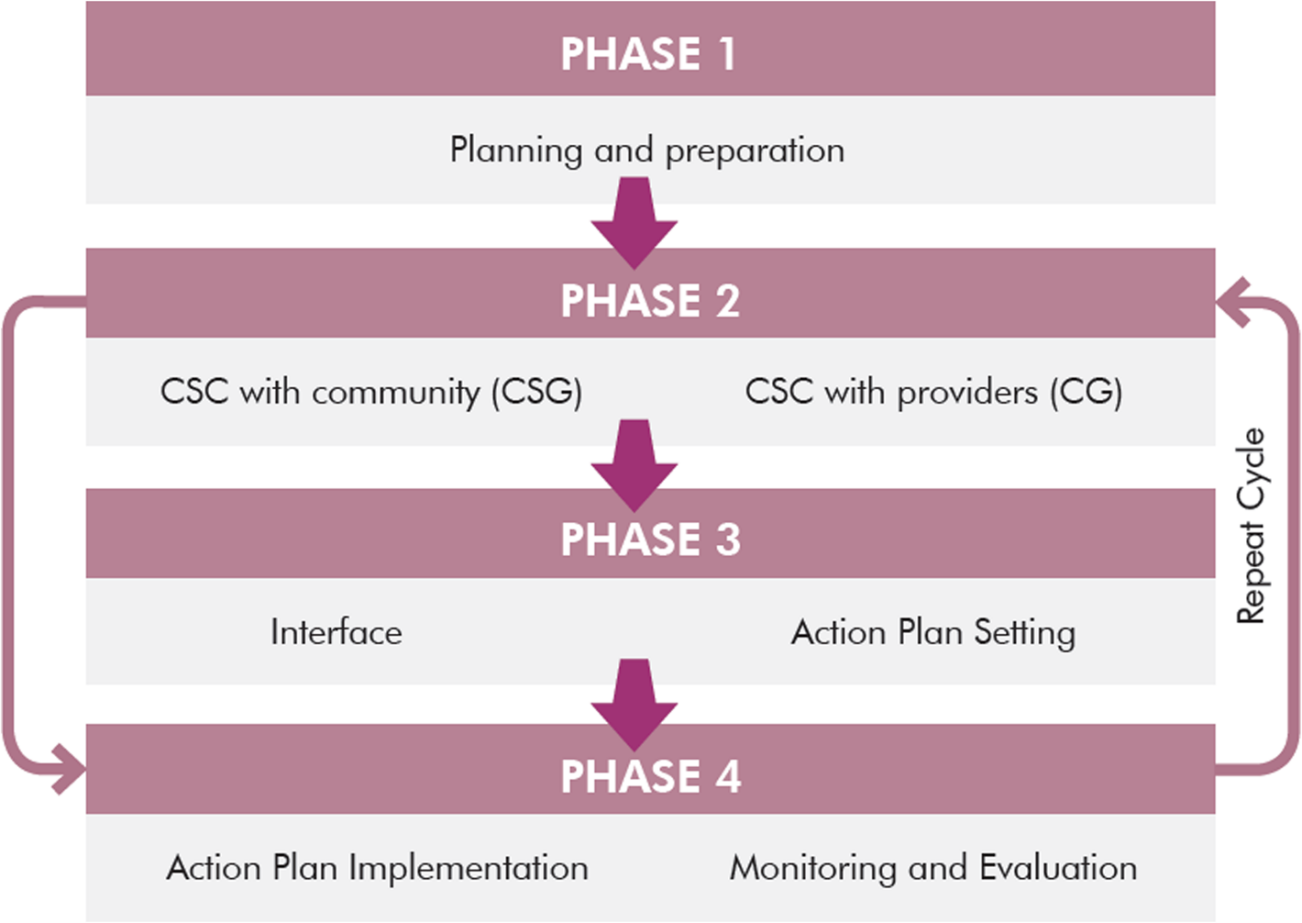
CSC implementation phases

The scorecard implementation followed four repeated phases: phase 1: planning and preparation with stakeholders from different levels; phase 2: community and provider generated scorecard sessions where both the groups separately identified issues and related indicators to improve on for their respective community clinics. The indicators were then scored on a scale of 1 to 5, 5 being “most satisfied” with the status of the indicators and 1 being “least satisfied”. Reasons behind scores were also recorded. Specific targets were then set to improve indicator scores within a specified period of time; phase 3: interface meeting between the providers and the community to come to a consensus about the priority indicators to work on and their scores and develop an action plan to reach set targets. The action plan included definite tasks, responsible person and required resources for each task; phase 4: action plan implementation and monitoring. Phase 1 to 4 was identified as 1 cycle of scorecard implementation and there was a 2 month gap between each cycle (<Fig. 1).

### Study design

A pilot study was conducted to explore feasibility and acceptability of community scorecard using qualitative research methods. The scorecard was implemented in Chakaria and Teknaf, two *upazilas* (sub-districts) in Cox’s Bazar district under the Chattogram division of Bangladesh. The sub-districts were purposively selected. icddr,b has its existence in both the areas. In Chakaria, icddr,b runs a health and demographic surveillance system since 1999. In Teknaf, icddr,b has been working on a community health project since 2013. This allowed operating the project on comparatively less resource and time duration. However, both the sub-districts are typical of rural Bangladesh and the public health facility setup is the same all around the country as in these two sub-districts. Chakaria has 23 CCs from where we purposively selected three: one in a remote area, one in a central area and one serving the tribal population of Chakaria, to allow for variation in context. Teknaf, on the other hand, had 12 CCs and we purposively selected one as intervention facility.

Data: 20 Key Informant Interviews (KII) and four Focus group Discussions (FGD) were conducted with purposively selected respondents (details of respondents in Table 1). The interviews and discussions were conducted by two research officers from icddr,b with background in anthropology. The qualitative data included information on knowledge about roles and responsibilities of members of CG and CSG, and accountability situation in health systems, knowledge about community scorecard, acceptance of community scorecard and the perceived benefits as well as challenges of implementing it. We tried to obtain the range of responses/observations in details from a sample of diverse informants. We arrived at the final sample size at the point of data saturation. Data was audio recorded, transcribed verbatim and was checked by the researchers who conducted the data collection to verify the content. Transcripts were coded using a coding scheme based on topics in the prepared guides and an initial reading of transcripts. Further, the analysis included observation data from 32 community representative group meetings, 12 provider group meetings, and 12 interface meetings taking place during the 3 cycles. Process documentation of scorecard implementation was also analysed. Desk review of community clinic’s operational manual and registry was conducted.

**Table 1:** Number of respondents for KII and FGDs

Type of respondent	no. of KII	Male	Female	Type of respondent	no. of FGDs	Male	Female
Local government representative	3	2	1	Community group	2 (14 members)	12	2
Healthcare provider	3	0	3	Provider group	2 (12 members)	8	4
Local elite	2	2	0				
Educated	4	2	2				
Illiterate	2	1	1				
Poor	2	1	1				
Adolescent	2	0	2				
Project facilitator	2	2	0				

### Definition of variables

#### Acceptability and feasibility

A combination of self-reported measures and observed behavioural measures were applied together to assess acceptability and feasibility of community scorecard. Factors such as participants’ attitudes towards the scorecard and its appropriateness, suitability, convenience and perceived effectiveness have been considered as indicators of acceptability. A modified version of the theoretical framework proposed by Sekhon et al. 2017 was used to assess acceptability in four domains. Feasibility, on the other hand, was defined in three domains [9] (Table 2).

**Table 2:** Domains of feasibility and acceptability of CSC

Theme	Domain	Qualitative theme (participants perspective)	Data source and method of data collection
Acceptability^a^	Affective attitude	General attitude towards CSC intervention	FGDs and KIIs with CG, CSG members, local govt. personnel, Project facilitator Observation data from meetings
	Self-efficacy	View on whether they can perform the behavior required to participate in CSC implementation.	
	Burden	View on whether amount of effort, time, human resource and cost required for CSC implementation and the opportunity cost of participation is acceptable	
	Perceived effectiveness	Perspective on usefulness and effectiveness of CSC in achieving set target	
Feasibility	Technical	View on adequacy of skill, technical competence of implementing staff at CCs	KII with CG,CSG members, local govt. personnel, Project facilitator
	Administrative	View on adequacy of staff at CC for CSC implementation	
	Financial	Opinion on whether fund for implementing CSC is available	

^a^adopted from [9]

## Results

### Acceptability of community scorecard

#### Affective attitude

The implementation of the scorecard was done in cycles and each cycle involved three meetings with community representative groups and one meeting with provider group in each CC. Participation rates at these scorecard sessions ranged between 80 to 85% for the community representative groups and between 96 to 100% for the provider groups. The combined participation of the community and the provider groups at the interface meetings ranged between 75 to 93%. Both the providers and the community saw the scorecard as a platform for constructive discussion around health in their locality. One key informant mentioned:

*“The local people are now more interested about CC. Earlier they did not think about CC much. Now they talk about the different challenges and try to solve them. They have meetings even without us to discuss these issues among themselves.” KII-14.*

All of the 18 KII respondents liked the scorecard process. The FGD participants were also in favor of scorecard. One of the key informants said:

*“We like the scorecard process very much - because it reveals the challenges we have in our area, in our CC, in our ward and within our community.”- KII-1.*

#### Self –efficacy

Understanding around the implementation of community scorecard was found to be improving among the participants over time. More than half of the respondents (10 out of 18) said they understood the process and would be able to implement it without any external support. On the other hand, there were also members who were yet to understand the whole implementation process. One KII respondent said:

*“We were told the maximum score for scorecard indicators was 5. What score would you give X indicator? I said I would give 5. Then they asked about Y indicator and asked (me) to score. I again said I would give 5. Then they (project personnel) explained saying, everyone graduates, someone gets 80 marks, someone gets 33 yet they both graduate. Does that mean all are same? Then I understood that I was doing it wrong. We were doing it wrong because we did not understand the process clearly. We then gradually understood how to differentiate the scores of each indicator.” KII-9.*

Some operational challenges were identified regarding understanding around scorecard implementation which included the risk of possible conflict of interest between the providers and the users. One of the meeting participants said:

*“Different people understand things differently. Sometimes they argue with each other. They end up marking indicators differently. There is no similarity among the group members. But then you have to help them to come to a consensus.”KII-4.*

#### Burden

The meetings of scorecard sessions were combined with the regular meetings of the community clinics as the objective of the scorecards complimented that of CC operations. The process included existing members of the CC management committees. Facilitation was mostly done by the project facilitators. The meeting participants felt that most of scorecard implementation is possible with existing resources. One member said: *“There are four committees in each CC. total … .. we have 68 members from the four committees and all are from the same area. One member can visit CC each day. This helps in monitoring”. KII-5.*

The chairman of CG committee mentioned that;

*“Scorecard serves the same purpose as that of our monthly meetings (CG, CSG meetings). But the process is useful - for example, this is a plus point in my meetings.” –KII-9.*

However, there are challenges in terms of financial resources required for meeting arrangements. As mentioned by one KII respondent:

*“Everyone goes to the meetings. They listen to the discussion. But when it comes to financial contribution, then some can contribute, the others cannot. They are ready to do everything apart from contributing financially. Because most of the people are very poor.” KII-10.*

#### Perceived effectiveness

According to the KII respondents, the community scorecard process was able to increase understanding and awareness around CC services among the committee members and the community people.

*“People are performing their duties around CC in between their other daily duties. People are doing this in their own way. This is all because scorecard has raised awareness among the committee members.” –KII-11.*

*“Community scorecard identified problems. When problems are identified, there will be initiatives to solve them, be it through the committee members, be it through the chairman. When everyone has these issues in their mind, there will be some improvement and we are already experiencing improvements.” -FGD-4.*

The respondents thought that collective effort and the presence of local elites in the scorecard process facilitated achievement of targets. They also expressed that the process allowed them to identify problems and prioritize them. Participants thought that community scorecard has been effective in improving communication between community and healthcare providers and also the behavior of healthcare providers towards the patients:

*“Before scorecard, patients used to complain about non availability of medicines. But now the list of available drugs is displayed at the CCs and people know which drug to expect.”-FGD-2.*

*“Yes, there has been a change in the behavior (of the provider). Previously they did not even want to talk to us! Now they treat us with respect, they talk to patients respectfully. When they (the providers) speak nicely with the patients and give good treatment, then they (the patients) are also happy and they (the patients) in turn, behave nicely too. This is definitely due to the scorecard” (FGD 1).*

Discussion around accountability and stakeholder relations during the planning phase resulted in prioritizing accountability issues in scorecard sessions by the participants. One of the key informants said:

*“The CC is supposed to be open from 9 am till 3 pm. But (the Community Health Care Provider [CHCP]) used to leave by noon/12:30 last year. Later this issue was brought up and discussed in meetings. Committee members were also aware about this situation since they pass the clinic, it’s on the road. After this (scorecard implementation), the clinic was open every day. Now, regardless of whether patients visit or not, the CHCP is present.” (FGD 1).*

However, according to the committee members and the project facilitators, the effectiveness of community scorecard was hampered by a few operational challenges. There were lack of awareness and orientation about roles and responsibilities among the committee members which hampered smooth and timely implementation of scorecard on the ground.

One of the project personnel mentioned:

*“Our primary challenge in implementing scorecard was to get the list of members of the CC committees. Even when we got the list, the names did not match in reality. As the meetings did not take place, no one used to monitor these.” KII-15.*

A healthcare provider also said: *“CSGs were not so active before. (People) did not come to the meetings, did not give much importance (to the committee). The scorecard, with support from icddr,b, has helped us in this regard.” KII-12.*

### Feasibility

#### Technical feasibility

During the planning phase from July 2017 till December 2017, a total of 32 mobilization sessions with 11 community representative groups and 12 sessions with 4 provider groups in the four intervention CCs were conducted. Participants were oriented on accountability in health service delivery and the major stakeholders in health sector, services provided at CCs, management of CC, their roles and responsibilities as CG and CSG members, community scorecard and its relevance in health, the steps to follow in implementing scorecard, time, effort and logistics required for the scorecard exercise. The second phase of each cycle of scorecard included a total of 11 community representative group meetings and 4 provider group meetings. Finally, in the third phase, for each cycle, four interface meetings were held for the four intervention CCs. The interface meetings identified 5–6 indicators per CC in cycle 1, 4–5 indicators per CC in cycle 2, and 3–4 indicators per CC in cycle 3.

Although the technicalities of scorecard implementation were followed by the participants, it required a basic level of facilitation from external sources. The implementation process was for a short period of time and during the 3 cycles of scorecard, the project facilitators could only hand over the facilitation skill partially. The participants were still having difficulty in understanding the construction of the scorecard matrix, developing indicators from problems, scoring them and finally prioritizing based on available resources and capacity. As mentioned by one of the project facilitator:

*“We have brought them to a stage where they will continue the meetings by themselves. But they might have difficulty in scoring and developing the format of scorecard without our facilitation. They still require some external support. However, they have become aware enough to discuss issues and identify problems.” KII-15.*

#### Administrative feasibility

The administrative support required for community scorecard implementation in each community clinic included alignment of scorecard objective with CC operational objectives, human resource for implementation, and the authority of CC management groups for scorecard implementation. Review of CC manual revealed that it includes a monitoring plan for clinics with involvement of CG and CSG. This is a strong support to continue the scorecard implementation as a complementary monitoring mechanism for CCs.

However, the process documentation data showed that although monitoring is embedded in current CC management, workload of the healthcare providers and lack of facilitation interrupts routine monitoring of the facilities. Further to this, data from FGDs revealed that the accountability mechanism in health system is weak and there is lack of effective monitoring from central level. The national health database, District Health Information Software 2 (DHIS2) only records the number of meetings and participants. No information on meeting outcome is recorded. The FGD participants mentioned:

*“We did come to the meetings before but did not talk at the meetings. We used to come to the meetings, have snacks, sign attendance sheet and leave. We did not know what our roles are and what our duties are (for CC). When did we get to know about all these? -When scorecard meetings started.”* FGD-3.

Many of the CSG members were included in the management group without their consent and they were reluctant to join the meetings and engage in any initiative around community clinics. In addition to these, political influence on choosing members for the two groups was a major constraint. Other members were in no position to hold these members responsible for any task or remove them from the committee. One CSG member mentioned:

*“Committee was formed by the chairman. He has included members of his choice. Now we can’t form a new committee even if we want. We will have to wait for the election after which we can talk to the chairman to form a better committee. Committee will need to be formed with active people.” KII-9.*

Observation data from meetings indicate lower participation and voice from some specific groups of members: women and adolescents. In particular, adolescent girls and boys were not always present at the meetings and even if they were, they were not vocal.

#### Financial feasibility

Community scorecard implementation required some additional funds to arrange regular meetings and follow up implemention of action plan. These included cost of tea, snacks, stationery, communication costs like phone bills, fuel for follow up activities. The existing CC fund generated from user fees is not enough to fund these activities.

The FGD participants expressed,

*“You need some money to arrange the meetings. There is an expense to arrange for snacks when 5/10 people sit together. You need to call meeting participants two to three times which has an expense. Apart from these, you need to buy a pen and other small things. But the clinic has no income. How much do you make by charging only TK.2 (as user fee). You will use it up in one meeting.”* FGD-1.

### Outcomes

**Fig. 2:**
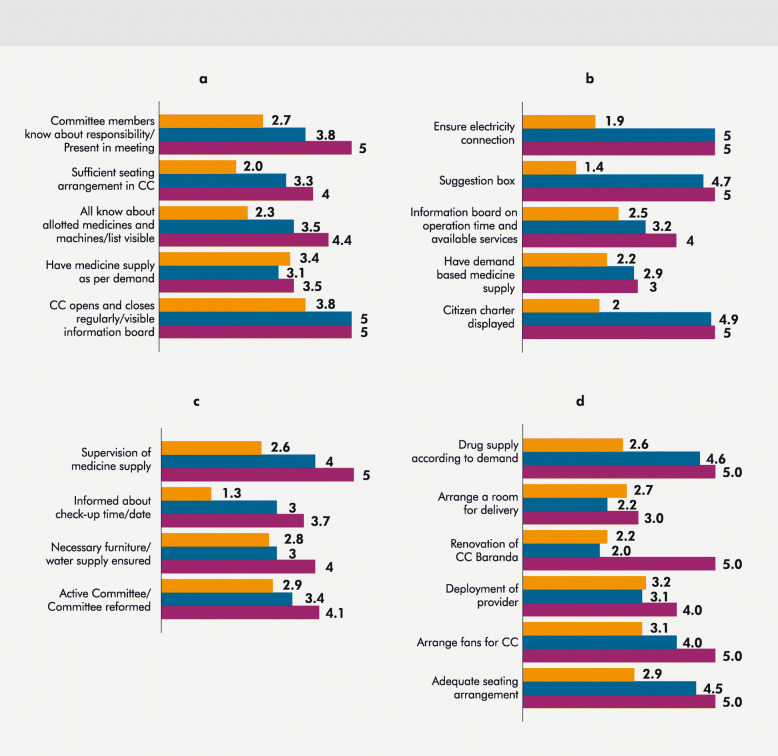
CSC indicators and changes during cycle 1, 2, 3. **a**: CSC indicators and changes in score, Manikpur CC. **b**: CSC indicators and changes in score, Baraitali CC. **c**: CSC indicators and changes in score, Shaharbil CC. **d**: CSC indicators and changes in score, Koyanchariapara CC, Teknaf. Cycle 1, Cycle 2, Cycle 3

During the 3 cycles over a period of 10 months, the scorecard process resulted in some positive changes in different dimensions including quality and accountability in health service delivery, community participation in health, revenue generation for health, raising community awareness. Key changes brought about by the scorecard process in the four community clinics are detailed in the following section and are presented in Fig. 2.

#### Community empowerment and community ownership

In Manikpur CC, there has been a steady increase in the score for committee members knowing about their roles and responsibilities and attending meetings (Fig. 2a). In Shaharbil CC, the community representatives made necessary reforms to committee formation and became more active over the period of the 3 cycles which is reflected through the increase in relevant indicator score over the 3 cycles (Fig. 2c).

#### Facility improvement and infrastructure management

After the problem of inadequate seating was identified in Manikpur CC in the first scorecard cycle, the provider group took the initiative to arrange for extra chairs with local donation resulting in increase in relevant indicator score (Fig. 2a). In Baraitali CC, lack of electricity was identified as a major problem and was scored only 1.9 in the first cycle. By Cycle 2, arrangements were made to ensure power supply, thereby achieving a score of 5 (Fig. 2b). In Shaharbil CC, the scorecard identified lack of water supply and inadequate furniture as problems where improvements were made over the 3 cycles (Fig. 2c). At Koyangchariapara CC of Teknaf, seating arrangement for clients was not adequate which was reflected by the average score of 2.9 in Cycle 1. With contribution from the community, the score increased to 5 in Cycle 3. The veranda of the community clinic was also renovated between cycle 1 and cycle 3. This CC also took the initiative to develop a delivery room at their premise which was one of the targets set in cycle 1 (Fig. 2d).

#### Ensuring accountability in service delivery

A major barrier in the community clinics was that the operation times of the CCs were not maintained properly. Providers were often absent or not punctual, and there was lack of clarity among users about what services are offered at the clinics. Improvements have been achieved in terms of maintaining regular clinic opening and closing times (Fig. 2a). In Baraitali CC, based on the indicators identified, the clinic committees installed a suggestion/complaint box, hung up an information board with operation times and available services and displayed the Citizen Charter at the facility premise, all of which are reflected in the increase in indicator scores over time (Fig. 2b). The score for supervision of medicine supply in Shaharbil CC has drastically improved to 5 since it was indicated as a problem in the first cycle and given a score of only 2.6 (Fig. 2c).

#### Community awareness about health services

In Shaharbil CC, the score for the indicator regarding awareness about antenatal/prenatal check-up times and dates increased from 1.3 in cycle 1 to 3.7 in cycle 3 (Fig. 2c). In Manikpur CC, the scores show that people’s knowledge about allotted medicines and machines in the clinic have improved over the 3 cycles (Fig. 2a).

## Discussion

The current paper is the first of its kind to document implementation feasibility and acceptability of community scorecard among both community members and healthcare providers in Bangladesh. So far the application of similar social accountability tools (e.g. community scorecard, citizen report card, participatory budgeting) in the country has been limited to non-health sectors [10]. The thematic framework analysis of feasibility and acceptability allowed understanding the multi-facet nature of community scorecard implementation in a low-resource setting.

The study showed that participants were enthusiastic and willing to take part in the scorecard process and they perceived the process to be useful in raising awareness about health in general in the community and facilitating structured monitoring of community clinic services. Providers viewed community scorecard as a complementary mechanism to the monitoring of CCs. The role of voice and collective action in improving health outcomes through scorecard were also appreciated. Studies in other parts of the world have also documented greater sense of community solidarity and partnership resulting from scorecard implementation [11].

The initial outcome of community scorecard implementation showed that the process was mostly influential in improving relationship between community and provider, creating an effective and inclusive space for negotiation between provider and user, increasing community participation in collective action to improve health service delivery, and availing local government fund in infrastructural improvement. The process built stronger community ownership and positively influenced accountability in health service delivery. Community scorecard also encouraged greater stakeholder engagement at different authoritative levels. Further, the process allowed systematically prioritizing issues around community clinic service provision, setting targets and SMART (specific, measurable, attainable, relevant and time-bound) indicators on supplies, operations, logistics, environment, and patient satisfaction. Several other countries around the world such as India, Ghana, Gambia, Kenya, Afghanistan, Democratic Republic of the Congo and Uganda have used community scorecards to tackle service delivery problems and have experienced improved accountability and transparency, enhanced availability, access, and quality of healthcare, improved community participation in health and stakeholder engagement [11–13]. A review of CARE’s community scorecard experience spanning over 10 years in 5 countries suggests that community scorecard has contributed to improvements in citizen empowerment, accountability and responsiveness, and expanded effective and inclusive spaces for negotiation between provider and user [13].

Despite the benefits of community scorecard, its implementation faced a few technical, administrative and contextual challenges. Some technical challenges in developing scorecards included conversion of issues to measurable indicators without external facilitation, short-term implementation limiting effect of scorecard, and time required to build facilitation skill among the users of scorecard. Lack of administrative and financial support to arrange regular meetings were also identified as implementation challenges. There is a lack of local government engagement in health sector in rural Bangladesh despite health being one of their priority mandates. The community clinic management committees should lobby to access resources earmarked for health in the local government fund. One of the major administrative barriers for scorecard implementation was the lack of effective monitoring from central level. Incorporating measurable outcome indicators for the meetings within the national health database DHIS2 can be considered to allow more effective monitoring. At the same time, the government of Bangladesh has recently launched a Multipurpose Health Volunteer cadre to be deployed at the community level to facilitate health service delivery [14]. Community scorecards, if implemented nationally, can benefit from this cadre of volunteers for additional monitoring support.

In terms of the scope of community scorecard, the tool had its limit in meeting targets that were beyond local level authority and for such issues, other complementary mechanisms might need to be identified. Further, in the context of Bangladesh, the community is largely unaware of their health rights and entitlements, and the providers do not always see accountability process as constructive. Conflict arising from difference in opinion among different parties was thus identified as challenge of scorecard implementation. The study showed that the initial mobilization and awareness building sessions can potentially play an effective role in neutralizing this gap in understanding. Some systems level factors were also identified which can impact effective implementation on the ground. The community scorecard intervention engaged the existing management committees of community clinics which had both community and provider representation making it possible to implement and scale up the process within the existing human resource setup. However, issues like political influence in selecting members and lack of voice of minorities in the group can eventually limit the effectiveness of community scorecard. For instance, absence or lack of voice of women and adolescent group can result in not giving due attention to health issues that are specific to these groups only.

The small scale implementation of community scorecard presented here was found feasible and acceptable for implementation within the national health system of Bangladesh. However, to establish its effectiveness on health outcomes and on improving accountability in health systems in low resource settings, the process needs to be tested on a larger scale and for a longer period of time. The findings identified a few issues that will be critical in ensuring sustainability of the process including building proper facilitation skills for scorecard sessions and identifying key facilitators, ensuring budget for meetings, and gradually institutionalizing the process in the operation of community clinics and other health facilities. At the policy level, the community scorecard process can be adopted to complement the existing monitoring mechanism of community clinics which is mandated by the government of Bangladesh [15].

Finally, the results presented here are based on implementation of community scorecard in four community clinics of Bangladesh for which the outcomes cannot be generalized for all of Bangladesh. The results are based on a very short term pilot implementation which does not allow capturing all the benefits or challenges of community scorecards. The implementation was mostly facilitated by the project personnel towards the beginning and even towards the end the community required some technical support from the project team. Without any external support the process might have taken longer.

## Conclusion

The results of the current study suggest that community scorecard is a feasible and acceptable tool to engage community and healthcare providers in monitoring and managing health facilities. Community scorecard has the potential to improve health service delivery at the community level, particularly for countries with health systems that are faced with challenges around accountability, quality and coverage of health services. The findings are intended to inform programme implementers, donors and other stakeholders about context, mechanisms, outcomes and challenges of scorecard implementation in Bangladesh and other developing countries. However, proper contextualization of the tool, institutional capacity building and policy integration will be critical in establishing its effectiveness at scale.

## Data Availability

Data and supporting material will be available following the Data Sharing Policy of icddr,b. All requests for data should be made to the corresponding author at shaila@icddrb.org

## Abbreviations

CSC: Community scorecard
CC: Community clinic
SMART: Specific, measurable, attainable, relevant and time-bound
CSG: Community Support Group
CG: Community Group
KII: Key Informant Interview
FGD: Focus Group Discussion
CHCP: Community Health Care Provider
DHIS2: District Health Information System, version 2
